# Retraction: The perception of Hungarian food by consumer segments according to food purchasing preferences based on primary research results

**DOI:** 10.1371/journal.pone.0321976

**Published:** 2025-03-31

**Authors:** The PLOS One Editors

After this article [1] was published, concerns were identified regarding the study design, reporting quality and conclusions, including the following:

- Insufficient detail is reported about the study design and methods (including participant recruitment, survey tool validation, definition of terms, and data analysis) preventing reproducibility, interpretation and assessment against community standards.
- There is a discrepancy between the information reported in the Methods section, which states “No race/ethnicity, age, disease/disabilities, religion, sex/gender, sexual orientation, or other socially constructed groupings related questions were raised”, and the demographic data reported in the Supporting Information file S1 Data.
- The reporting of the results is incomplete and insufficiently clear. The Supporting Information includes only a subset of data collected for the quantitative analysis, without providing details of how the responses are coded, and the qualitative study results and secondary source analysis results are not provided or discussed.
- The article presents statements and conclusions that are not supported by the study design and results.

The corresponding author stated that gender and age were requested but that they did not ask about the race, sexuality, or religion of the respondents.

Following editorial assessment and consultation with a member of the journal’s Editorial Board, the *PLOS One* Editors concluded that the article does not meet the journal’s criteria for publication.

In light of the above concerns, the *PLOS One* Editors retract this article [1]. We regret that the issues were not identified prior to the article’s publication.

All authors did not agree with the retraction.
